# Lower serum calcium levels are a risk factor for a decrease in eGFR in a general non-chronic kidney disease population

**DOI:** 10.1038/s41598-018-32627-4

**Published:** 2018-09-21

**Authors:** Satoru Mizushiri, Makoto Daimon, Hiroshi Murakami, Aya Kamba, Sho Osonoi, Masato Yamaichi, Koki Matsumura, Jutaro Tanabe, Yuki Matsuhashi, Miyuki Yanagimachi, Itoyo Tokuda, Shizuka Kurauchi, Kaori Sawada

**Affiliations:** 1 Department of Endocrinology and Metabolism, Hirosaki University Graduate School of Medicine, Hirosaki, Aomori, Japan; 2 Department of Social Medicine, Hirosaki University Graduate School of Medicine, Hirosaki, Aomori, Japan

## Abstract

Association between serum calcium (Ca) levels and kidney dysfunction progression in a non-chronic kidney disease (CKD) population has not been well elucidated, especially in consideration for classical metabolic risk conditions such as hypertension, dyslipidemia, and diabetes, and those related to Ca metabolism. Among participants of the population-based Iwaki study of Japanese people, those with an estimated glomerular filtration rate (eGFR) ≧60 ml/min/1.73 m^2^ and age ≧40 years, and who attended the study consecutively in 2014 and 2015 were enrolled (gender (M/F): 218/380; age: 58.9 ± 10.2). Regression analysis showed a significant correlation between serum Ca levels and a change in eGFR in the 1-year period (∆eGFR) after adjustment with multiple factors including those related to Ca metabolism (β = 0.184, p < 0.001). When subjects were stratified into tertiles based on their serum Ca levels (higher >9.6 mg/dL, middle 9.4–9.6 mg/dL, lower <9.4 mg/dL), lower serum Ca levels were a significant risk for a rapid decliner of eGFR designated as the lower one third of ∆eGFR (<−4.40 ml/min/1.73 m^2^) (odds ratio 2.41, 95% confidence interval 1.47–3.94). Lower serum Ca levels are a significant risk for rapid decrease in eGFR, independent of previously reported metabolic risk factors in this general population with non-CKD, or eGFR ≧60 ml/min/1.73 m^2^.

## Introduction

Chronic kidney disease (CKD) is a worldwide public health issue, because CKD is a risk for cardiovascular events and progression of kidney failure and, according to world health organization global health estimates, 1.5% of deaths worldwide were attributable to this condition in 2012^1,2^. Therefore, prevention of the progression of kidney dysfunction in those with CKD seems to be very important. For this purpose, various studies have examined factors for predicting progression of kidney dysfunction and, thus, seem to be useful to find those should be treated^1–6^. Metabolic conditions such as hypertension, dyslipidemia, hyperuricemia and diabetes are factors that cause kidney dysfunction^1,2,7–10^. However, because CKD is a consequence of not just one cause but various conditions that alter the function and structure of the kidney irreversibly, such factors may vary depending on background conditions and/or the severity of CKD. To date, most studies have focused on finding such factors in patients with CKD, or estimated Glomerular Filtration Rate (eGFR), <60 mL/min/1.73 m^2^ (CKD stages 3 and more). However, because kidney function appears to decrease continuously, though the extent may be less, even before it reaches the stage 3 of CKD, it may be also valuable to examine factors associated with a decrease in eGFR in those without CKD stages 3 and more.

Although various metabolic conditions such as hypertension, dyslipidemia, hyperuricemia and diabetes are associated with kidney dysfunction, the association between other common factors such as serum calcium (Ca) levels and kidney dysfunction have not been well elucidated, especially when considered with the effects of the above-mentioned metabolic conditions and in those without CKD stages 3 and more. Abnormalities in serum Ca levels affect neurological, gastrointestinal and kidney functions. Severe hypercalcemia and/or rapidly rising serum Ca levels cause a reduction in eGFR^11^. In patients with primary hyperparathyroidism, hypercalcemia causes renal dysfunction by multiple mechanisms such as hypercalciuria, nephrolithiasis, and nephrocalcinosis^12^. Further, overtreatment for hypocalcemia caused by hypoparathyroidism induces kidney dysfunction by hypercalciuria, because its standard treatment is not parathyroid hormone (PTH) replacement but supplementation of vitamin D analogs and Ca^13^. Therefore, abnormal serum Ca levels appear to be associated with progression of kidney dysfunction. However, the association between serum Ca levels within a physiological range and progression of kidney dysfunction has not been well examined so far.

Here, we examined the association between serum Ca levels within a physiological range and change in kidney function or eGFR in a non-CKD (CKD stages 1 and 2) general Japanese population, in consideration with multiple factors known to be associated with kidney function, and also with those involved in Ca metabolism (i.e. serum levels of inorganic phosphorus (InP), PTH, and 1,25-dihydroxyvitamin D (VitD)). The study suggests that serum Ca levels are a useful marker to predict those at risk for progression of kidney dysfunction in those without CKD.

## Material and Methods

### Study Population

Subjects were recruited from the Iwaki study, a health promotion study of Japanese people over 20 years of age that aims to prevent lifestyle-related diseases and prolong lifespans. The study is conducted annually in the Iwaki area of the city of Hirosaki in Aomori Prefecture in northern Japan^14,15^. To evaluate the decrease in eGFR in 1 year, those who attended the Iwaki study consecutively in 2014 and 2015 were enrolled in this study. Stage of CKD was defined base on Kidney disease: improving global outcomes (KDIGO) classification^16^. Of the 806 such individuals, those with eGFR <60 ml/min/1.73 m^2^ (n = 62; the prevalence of CKD was 7.7/%) were excluded because they were considered as having CKD (stage 3 and more). Those who were under 40 years old (n = 146) were also excluded to avoid the effects of differences in age-dependent changes in GFR between subjects under and over 40 years old: Age-dependent linear decreases in GFR were reported in subjects over 40 years old, with no changes in GFR in subjects under 40 years old^17,18^. After these exclusions, 598 individuals (218 men, 380 women) aged 58.9 ± 10.2 years were included in the study. Although only CKD stage 1 is defined as normal, we here arbitrary used the term “non-CKD” for CKD stage 1 and 2 combined to explain clearly the differences in the study design between the current study and others examining factors associated with decreases in kidney function, most of which used subjects with CKD stages 3 and more as those with CKD.

This study was approved by the Ethics Committee of the Hirosaki University School of Medicine (No. 2014-014 and 2014-015), and was conducted according to the recommendations of the Declaration of Helsinki. Written informed consent was obtained from all participants.

### Characteristics Measured

Blood samples were collected in the morning from peripheral veins of participants under fasting conditions in a supine position for 5 min after 10 min rest in a sitting position. The following clinical characteristics were measured: height, body weight, body mass index, percent body fat (fat), fasting blood glucose, fasting serum insulin levels, glycated hemoglobin (HbA1c), systolic and diastolic blood pressures, serum levels of total cholesterol, triglyceride, high-density lipoprotein-cholesterol, uric acid, urea nitrogen, creatinine (Cr), Na, K, Ca, InP, intact PTH, and VitD, and urine albumin excretion (UAE). Fat was measured by the bioelectricity impedance method using a Tanita MC-190 body composition analyzer (Tanita Corp., Tokyo, Japan). HbA1c (%) is expressed as the National Glycohemoglobin Standardization Program value. All laboratory testings were performed in a commercial laboratory (LSI Medience Co., Tokyo, Japan) according to the instructions of the vendors. Serum Ca levels were corrected based on serum albumin levels as previously reported^19^. Estimated GFR (eGFR) was calculated by following formulas published by the Japanese Society of Nephrology, eGFR = 194 × Cr^−1.094^ × Age^−0.287^ (Man) or 194 × Cr^−1.094^ × Age^−0.287^ × 0.739 (Woman)^20^. Diabetes was defined according to the 2010 Japan Diabetes Society criteria, i.e. FBG concentrations ≧126 mg/dl^21^. In subjects whose FBG levels were not measured, diabetes was defined as HbA1c levels ≧6.5%. None of the subjects in our study were known to have type 1 diabetes. Hypertension was defined as blood pressure ≧140/90 mmHg or being treated for hypertension (n = 286). Hyperlipidemia was defined as total cholesterol ≧220 mg/dl, TG ≧ 150 mg/dl or being treated for hyperlipidemia. (n = 280). Drinking alcohol (current or non-drinker) and smoking habits (never, past or current) were determined from questionnaires.

### Statistical analysis

Clinical characteristics are given as mean ± standard deviations (SD). The statistical significance of the difference in characteristics values between two groups (parametric) and case-control associations between groups (nonparametric) were assessed by analysis of variance and χ^2^ tests, respectively. For UAE (data were shown as median with interquartile range (IQR)), Wilcoxon signed-rank test was used to determine the statistical significance. Correlations between ∆eGFR (change in eGFR in the 1 year period) and clinical characteristics were assessed by linear regression analyses. Risk of lower serum Ca levels for decreased eGFR was calculated by multiple logistic regression analysis with adjustment for factors found to be associated with ∆eGFR by univariable regression analysis as well as factors well known to be related to a decrease in eGFR and/or kidney function. Besides, age, gender, and serum levels of Cr were not included as covariables for analyses using eGFR as a dependent variable, since these factors are used for calculation of eGFR per se. Receiver operating characteristic (ROC) curve analysis was performed to determine a cut-off value of the serum Ca levels to predict decreased eGFR. The statistical power to detect the differences in the frequencies of the rapid decliner of eGFR estimated using the software Sampsize (http://sampsize.sourceforge.net/iface/index.html) in this sample set were about 80% and 40% to detect minimal ORs of 2.00 and 1.50, respectively, at a level of significance of 0.05. A value of p < 0.05 was accepted as statistically significant. All analyses were performed using SPSS version 23.0 (IBM Japan, Tokyo, Japan) or JMP version 12.0 (SAS Institute Japan Ltd., Tokyo, Japan).

## Results

### Clinical Characteristics of The Study Subjects

The clinical characteristics of subjects at baseline and follow-up are shown in Table 1. Most characteristics changed significantly in the 1-year period, as eGFR decreased significantly from 80.41 ± 11.96 to 78.55 ± 11.26 (p < 0.001). However, serum Ca levels did not change in the period: means ± SD at the baseline and the follow-up were 9.47 ± 0.31 and 9.48 ± 0.31, respectively (medians [IQR] were 9.5 [9.3–9.7] and 9.5 [9.3–9.7], respectively) (p = 0.21).

**Table 1:** Clinical characteristics of the subject at baseline and follow-up.

Characteristics	Baseline	Follow-up	p
Gender (M/W)	218/380	218/380	NA
Age (yr)	58.9 ± 10.2	59.9 ± 10.2	<0.01**
Height (cm)	159.2 ± 8.7	159.1 ± 8.7	<0.01**
Body weight (kg)	58.4 ± 10.6	58.2 ± 10.6	0.02*
Body mass index (kg/m^2^)	22.9 ± 3.1	22.8 ± 3.6	0.08
Fat (%)	25.4 ± 8.0	25.7 ± 8.0	<0.01**
Fasting plasma glucose (mg/dL)	83.4 ± 14.0	83.6 ± 13.6	0.66
HbA1c (%)	5.79 ± 0.50	5.79 ± 0.51	0.54
Fasting serum insulin (μU/mL)	4.59 ± 4.30	4.61 ± 4.71	0.81
Systolic blood pressure (mmHg)	130.9 ± 19.3	123.5 ± 17.3	<0.01**
Diastolic blood pressure (mmHg)	78.9 ± 10.8	75.5 ± 11.3	<0.01**
Total cholesterol (mg/dL)	204.0 ± 30.8	211.4 ± 32.4	<0.01**
Triglyceride (mg/dL)	93.4 ± 67.6	96.3 ± 64.4	0.20
LDL Cholesterol (mg/dL)	119.3 ± 27.7	120.9 ± 28.0	0.04*
HDL Cholesterol (mg/dL)	65.8 ± 16.5	67.8 ± 17.4	<0.01**
Serum albumin (g/dL)	4.46 ± 0.25	4.47 ± 0.28	0.34
Serum uric Acid (mg/dL)	4.75 ± 1.25	4.83 ± 1.25	<0.01**
Serum urea Nitrogen (mg/dL)	15.07 ± 3.99	15.17 ± 3.83	0.45
Serum creatinine (mg/dL)	0.66 ± 0.12	0.67 ± 0.12	<0.01**
eGFR (ml/min/1.72 m^2^)	80.41 ± 11.96	78.55 ± 11.26	<0.01**
UAE (median [IQR]) (mg/gCre)	10.5 [6.7–18.3]	7.6 [4.7–13.8]	<0.01**
Serum Na (mmol/L)	142.0 ± 1.75	141.6 ± 1.76	<0.01**
Serum K (mmol/L)	3.83 ± 0.30	3.88 ± 0.32	<0.01**
Serum Cl (mmol/L)	104.3 ± 1.8	103.7 ± 2.1	<0.01**
Serum Ca (mg/dL)	9.47 ± 0.31	9.48 ± 0.31	0.21
Serum InP (mg/dL)	3.50 ± 0.45	3.47 ± 0.43	0.04*
intact PTH (pg/mL)	51.9 ± 15.9	51.9 ± 16.2	0.97
1,25-dihydroxyvitamin D (pg/mL)	65.4 ± 24.7	ND	—
Hypertension: n (%)	286 (47.8)	259 (43.3)	<0.01**
Hyperlipidemia: n (%)	280 (46.8)	321 (53.7)	<0.01**
Diabetes: n (%)	55 (9.2)	53 (8.9)	0.66
Drinking alcohol: n (%)	253 (42.3)	246 (41.3)	<0.01**
Smoking (Never/Past/Current): n	386/122/90	396/114/86	0.19

P < 0.05 and <0.01 are indicated by * and **, respectively. Data are mean ± SD or number of subjects (%). eGFR: estimated Glomerular Filtration Rate, UAE: Urinary albumin excretion, In P: inorganic phosphorus, PTH: parathyroid hormone, IQR: interquartile range, ND: not determined.

### Factors Correlated With Changes in eGFR in The 1-year Period (∆eGFR)

Correlations between clinical characteristics and ∆eGFR are shown in Table 2. Univariable correlation analyses revealed correlations between ∆eGFR and multiple clinical characteristics such as serum levels of LDL-cholesterol, uric acid, creatinine, sodium (Na), Ca, and VitD. However, multivariable correlation analysis revealed serum Ca levels as the best correlated with ∆eGFR after adjustment with multiple factors observed as correlated by univariable analyses and well-annotated classical risk factors for a decrease in eGFR such as hypertension, dyslipidemia (serum triglyceride and LDL-cholesterol levels were included), and diabetes (β = 0.184, p < 0.001). Although the classical risk factors examined were not at all correlated with ∆eGFR in this study population, the correlation of serum Ca levels with ∆eGFR independent of such classical risk factors observed in ordinary clinical settings is summarized together with those of several clinical characteristics related to kidney function (urinary albumin excretion ratio (UAE), serum levels of potassium (K), InP, intact PTH, and VitD) in Table 3.

**Table 2:** Factors correlated with changes in eGFR in the 1-year period (∆eGFR).

Characteristics	Univariable		Multivariable	
	β	p	β	p
Height (cm)	−0.023	0.58	—	—
Body weight (kg)	−0.056	0.17	—	—
Body mass index (kg/m^2^)	−0.056	0.17	−0.050	0.26
Fat (%)	−0.050	0.22	—	—
Fasting plasma glucose (mg/dL)	−0.023	0.58	—	—
HbA1c (%)	−0.021	0.61	—	—
Fasting serum insulin (μU/mL)	0.022	0.59	—	—
Systolic blood pressure (mmHg)	−0.014	0.72	—	—
Diastolic blood pressure (mmHg)	0.014	0.73	—	—
Total cholesterol (mg/dL)	0.061	0.13	—	—
Triglyceride (mg/dL)	−0.050	0.22	−0.066	0.13
LDL Cholesterol (mg/dL)	0.087	0.03*	0.039	0.34
HDL Cholesterol (mg/dL)	−0.001	0.97	—	—
Serum albumin (g/dL)	0.076	0.06	—	—
Serum uric Acid (mg/dL)	0.101	0.01*	0.079	0.08
Serum urea Nitrogen (mg/dL)	0.026	0.53	—	—
Serum creatinine (mg/dL)	0.265	<0.01**	—	—
UAE (mg/gCre)	−0.022	0.60	0.001	0.097
Serum Na (mmol/L)	0.149	<0.01**	0.145	<0.01**
Serum K (mmol/L)	0.064	0.12	0.047	0.26
Serum Cl (mmol/L)	−0.064	0.12	—	—
Serum Ca (mg/dL)	0.212	<0.01**	0.184	<0.01**
Serum InP (mg/dL)	0.046	0.26	0.001	0.99
Intact PTH (pg/mL)	−0.025	0.54	0.012	0.77
1,25-dihydroxyvitamin D (pg/mL)	−0.084	0.04*	−0.086	0.04*
Hypertension: n (%)	−0.024	0.55	0.019	0.66
Hyperlipidemia: n (%)	0.015	0.72	—	—
Diabetes: n (%)	−0.038	0.35	0.046	0.26
Drinking alcohol: n (%)	−0.027	0.52	—	—
Smoking (Never/Past/Current):n	−0.020	0.62	—	—

P < 0.05 and <0.01 are indicated by * and **, respectively. Data are mean ± SD or number of subjects (%). eGFR: estimated Glomerular Filtration Rate, UAE: Urinary albumin excretion, InP: inorganic phosphorus, PTH: parathyroid hormone.

**Table 3:** Correlation of risk factors with ∆eGFR.

	Univariable		Model 1		Model 2	
	β	p	β	p	β	p
Serum Ca (mg/dL)	0.212	<0.01**	0.190	<0.01**	0.184	<0.01**
Serum InP (mg/dL)	0.046	0.26	0.044	0.29	0.001	0.99
intact PTH (pg/mL)	−0.025	0.54	−0.033	0.42	0.012	0.77
VitD (pg/ml)	−0.084	0.04*	−0.080	0.05	−0.086	0.04*
Serum K (mmol/L)	0.064	0.12	0.067	0.10	0.047	0.26
UAE (mg/gCre)	−0.022	0.60	−0.006	0.89	0.001	0.97

P < 0.05 and <0.01 are indicated by * and **, respectively. Adjusted for serum levels of LDL-cholesterol, triglyceride, uric acid, and Na, hypertension, and diabetes (Model 1) + serum levels of InP, K intact PTH, and VitD, BMI, and UAE (Model 2). VitD: 1,25-dihydroxyvitamin D, UAE: Urinary albumin excretion, InP: inorganic phosphorus, PTH: parathyroid hormone.

### Association of Lower Serum Ca Levels and a Decrease in eGFR

**Figure 1:**
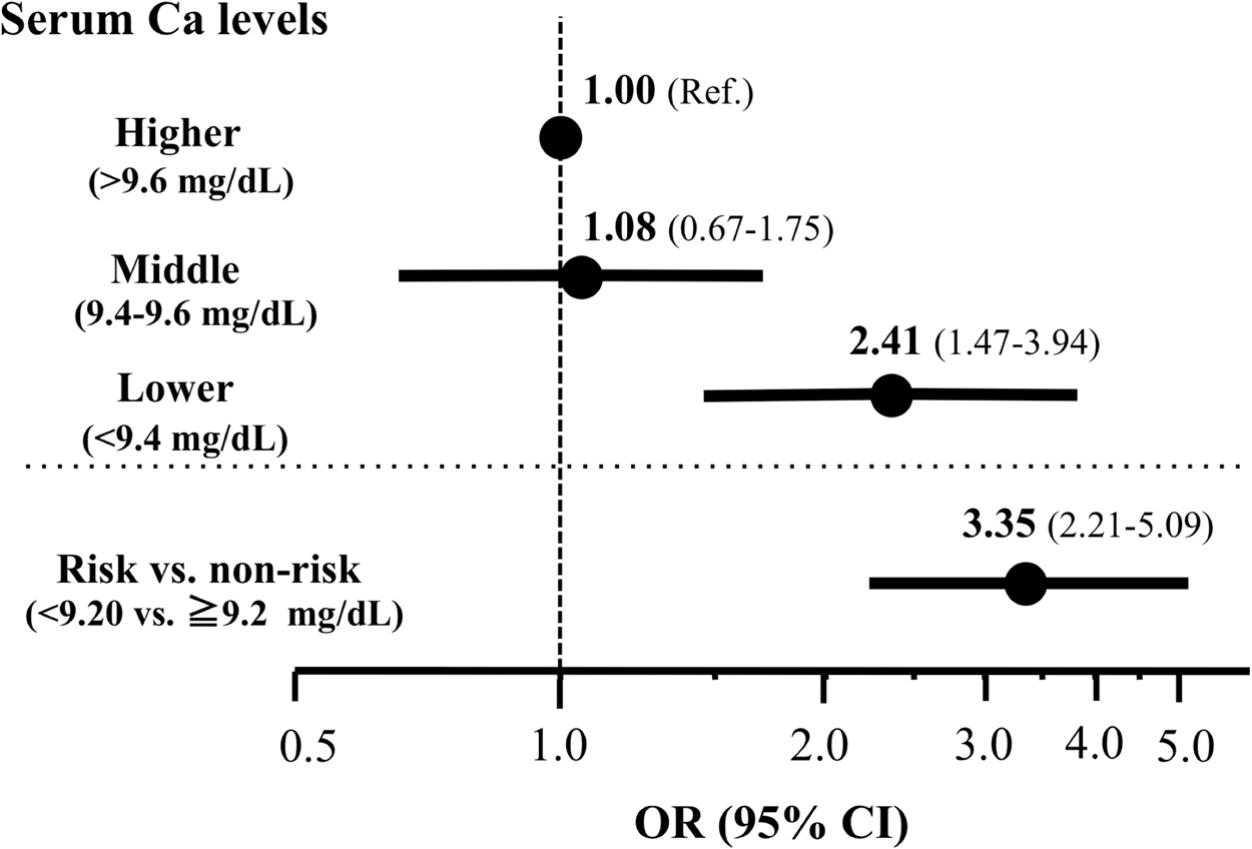
Risk for a rapid decliner of eGFR. Odds ratio (OR)s with 95% confidence interval (CI) are shown. Adjusted for multiple factors: serum levels of LDL cholesterol, triglyceride, uric acid, Na, Ca, InP, K, intact PTH, and VitD, BMI, hypertension, diabetes, and UAE. Ref: reference, VitD: 1,25-dihydroxyvitamin D, UAE: Urinary albumin excretion, InP: inorganic phosphorus, PTH: parathyroid hormone.

To further evaluate the relationship between serum Ca levels and a decrease in eGFR, subjects were stratified into tertiles based on their serum Ca levels (higher >9.6 mg/dL, middle 9.4–9.6 mg/dL, lower <9.4 mg/dL). We then evaluated the risks of these tertiles for a rapid decliner, which we tentatively designated as those with the lower one third of ∆eGFR (<−4.40 ml/min/1.73 m^2^). Those with the lower serum Ca levels were on a significant risk for a rapid decliner of eGFR compared to those with the higher serum Ca levels (odds ratio (OR): 2.55, 95% confidence interval (CI): 1.63–3.99). The risk remained significant after adjustment for the multiple factors described above (OR 2.41, 95% CI 1.47–3.94). ROC analyses showed a serum Ca level of 9.20 mg/dl as an optimal cut-off value for determining the risk subjects for a rapid decliner of eGFR (OR: 3.35, 95% CI: 2.21–5.09). Together, these results indicate that lower serum Ca levels, even when they are within normal range, are a significant risk factor for a rapid decrease in eGFR, independent of previously reported metabolic risk factors for kidney dysfunction such as blood pressure, dyslipidemia, and diabetes, in this general non-CKD Japanese population (Fig. 1).

## Discussion

In this 1-year follow-up study of a general non-CKD Japanese population, we found that lower serum Ca levels, even when they were within a normal physiological range, were a significant risk factor for a rapid decrease in eGFR, independent of previously reported metabolic risk factors for kidney dysfunction such as blood pressure, dyslipidemia, and diabetes. Further, the fact that such metabolic risk factors were not associated with ∆eGFR indicates that such metabolic risk factors can be used to predict those at risk for worsening kidney function only after kidney function has declined to certain levels, such as those of CKD. Instead, lower serum Ca levels appear to be the most useful marker to predict those at risk for worsening kidney function while kidney function is normal or only mildly decreased.

Serum Ca levels are regulated through feed-back mechanisms, where PTH, VitD and serum ionized Ca itself coordinate to regulate Ca transport at the gut, kidney, and bone^22^. Thus, any abnormality in the mechanisms might affect the results obtained here. However, because the subjects used here were apparently healthy and we used serum levels of PTH and VitD for adjustment, the results obtained here did not appear to merely reflect such an abnormality of Ca metabolism.

Association does not indicate a cause and effect relationship, by the nature of the analysis. Therefore, the observed association between lower serum Ca levels and a rapid decrease in eGFR dose not simply mean that lower serum Ca levels impair kidney function, or, conversely, that lower serum Ca levels are a consequence of a rapid decrease in eGFR. However, as described previously, since serum Ca levels are in cause-consequence relationships with several factors such as serum level of InP, VitD, and i-PTH, we adjusted for such factors, and also K and UAE, whose increases are markers of existing kidney dysfunction. Thus, lower serum Ca levels appear to not be simply a reflection of existing or current kidney dysfunction, but a reflection of a steady or rapid decrease in eGFR. Together, lower serum Ca levels seem to be a useful marker for finding those at-risk for worsening kidney function in a general population or in ordinary clinical settings. We here examined the association between serum Ca levels and a decrease in kidney function in those with CKD stages 1 and 2 combined, on the assumption that kidney function decreases similarly between these 2 CKD stages. However, since decreases in kidney function may differ between these 2 CKD stages, the association observed may also differ between these 2 CKD stages. To answer the question, we examined the association using the subjects with CKD stages 1(n = 125) and 2 (n = 473) separately, and, found no different results between them: univariable linear correlation between serum Ca levels and ∆eGFR were 0.205, p = 0.02 and 0.144, p < 0.01, in the analyses using those with CKD stages 1 and 2, respectively. Further, since Ca homeostasis may be deregulated little bit even in CKD stage 2, serum Ca levels may, even though within the normal range, fluctuate more in those with CKD stage 2 than in those with CKD stage 1. However, since serum Ca levels and their SD (or fluctuation) were 9.36 and 9.49 (p < 0.01), and 0.36 and 0.29 (p < 0.01), respectively, in those with CKD stages 1 and 2, respectively, regulation of Ca homeostasis did not appear to be substantially deteriorated in those with CKD stage 2. These facts together indicated that, as far as serum Ca levels were concerned in its association with ∆eGFR, difference between CKD stages 1 and 2 might not have a substantial impact on its association. Anyway, since subjects with CKD stages 1 and 2 are of course a mixture of a variety of subjects with different compensatory mechanisms where Ca, InP, and VitD also act cooperatively for maintaining the homeostasis along with declining kidney function, further detailed analyses using subjects with CKD stages 1 and 2 separately appear to be warranted in the future.

Although not many studies examined factors associated with changes in eGFR in general population similar to our study, a study showed urine volume to be inversely associated with annual decline of eGFR in those with normal kidney function, which they defined as those with eGFR ≧ 60 mL/min/1.73 m^2^ ^23^. Interestingly, their findings were opposite to those found in another observational study with CKD patients, where higher urine volume was associated with faster decline in kidney function^24^. These facts appear to indicate that association of any factors with ∆eGFR may differ depending on stage of CKD. In this context, findings observed in CKD patients (CKD stages 3 and more) may not be applied in patients without CKD (or with CKD stages 1 and 2, non-CKD, or normal kidney function), and, thus, these facts seem to strengthen the importance of findings reported here, especially, provided that population of those without CKD are generally much higher than that with CKD.

The results appear to warrant further studies examining whether intervention to increase serum Ca levels is useful in preventing worsening kidney dysfunction. However, supplementation of Ca does not seem to be such an intervention. In CKD subjects, where Ca balance and homeostasis are disrupted, supplementation of Ca may enhance soft tissue calcification and cardiovascular diseases, and thus may be harmful^22,25^. In non-CKD, or healthy subjects, in whom Ca balance and homeostasis are tightly regulated, serum Ca levels do not seem to change easily upon supplementation of Ca, and thus supplementation of Ca does not seem to be useful for increasing serum Ca levels. Indeed, the amounts of Ca consumed evaluated base on a dietary questionnaire (BDHQ) was not correlated with serum Ca levels in the study population (β = 0.03, p = 0.40). Supplementation of VitD seems to be an option to prevent worsening kidney dysfunction, because VitD has been shown to prevent kidney damage and a decrease in GFR in experimental models through various mechanisms. These include activation of the renin-angiotensin-aldosterone system, reduction of inflammatory mediators, promotion of survival of podocytes, and reduction of albuminuria and glomerulosclerosis^26–31^. However, the effects of VitD supplementation on human kidney function have not yet been well elucidated, although supplementation of VitD is established for management of the osteomalacic component of CKD-related mineral and bone disorders^21^. A study using older adults with predominantly normal baseline kidney function showed an association between lower serum VitD levels and rapid decline of GFR^32^, which appeared to indicate promising effects of supplementation of VitD on kidney function in a general population. However, in the current study, higher serum VitD levels were found to be associated with a decrease in eGFR, although the extent was modest (β = −0.084, p = 0.048). Therefore, the effects of VitD supplementation on kidney function in a general population remains to be elucidated. Our study has both strengths and limitations. For strengths, statistical adjustments were made for multiple factors that could confound the results, and a relatively large population-based/general sample of individuals was used. Most importantly, we used a general population where kidney function is normal or only mildly decreased, and we could thus examine the association between serum Ca levels and changes in eGFR in a non-CKD population. For limitations, the subjects were participants in a health promotion study rather than an ordinary health check-up study, and, thus, may be more invested in keeping themselves healthy compared with the general population. Thus, the subjects may not accurately represent the general population. Further, we followed up on changes in eGFR in 1-year period, which appears to be not long enough to evaluate time-dependent changes in eGFR precisely. Because the changes in eGFR in 1-year period appear to be modest compared with the deviation of measurements of eGFR per se, the statistical power to evaluate time-dependent changes in eGFR appears to be low. However, even in this condition, we could find a significant association between serum Ca levels and changes in eGFR, and such limitation therefore does not seem to be substantial. In conclusion, lower serum Ca levels, even within a normal physiological range, were a significant risk factor for a rapid decrease in eGFR in a non-CKD Japanese population, independent of previously reported metabolic risk factors for kidney dysfunction such as blood pressure, dyslipidemia, and diabetes. These results suggest that lower serum Ca levels can be a useful marker to predict those at-risk for worsening of kidney function in ordinary clinical settings, and, thus, further studies using cohort populations with longer follow-up periods are warranted.

## References

[1] Go AS, Chertow GM, Fan D, McCulloch CE, Hsu CY (2004). Chronic kidney disease and the risk of death, cardiovascular events, and hospitalization. N Engl J Med.

[2] Webster AC, Nagler EV, Morton RL, Masson P (2017). Chronic Kidney Disease. Lancet.

[3] Voormolen N (2007). PREPARE study group: High plasma phosphate as a risk factor for decline in renal function and mortality in pre-dialysis patients. Nephrol Dial Transplant.

[4] Staples AO (2010). Association between clinical risk factors and progression of chronic kidney disease in children. Clin J Am Soc Nephrol.

[5] Peters KE (2017). Identification of Novel Circulating Biomarkers Predicting Rapid Decline in Renal Function in Type 2 Diabetes: The Fremantle Diabetes Study Phase II. Diabetes Care.

[6] Leiherer A (2018). The value of uromodulin as a new serum marker to predict decline in renal function. J Hypertens.

[7] Lewis EJ, Hunsicker LG, Bain RP, Rohde RD (1993). The effect of angiotensin-converting-enzyme inhibition on diabetic nephropathy. N Engl J Med.

[8] Barkis GL (2000). Preserving renal function in adults with hypertension and diabetes: A consensus approach. Am J Kidney Dis.

[9] Kang DH (2002). A role for uric acid in the progression of renal disease. J Am Soc Nephrol.

[10] Manttari M, Tiula E, Alikoski T, Manninen V (1995). Effects of hypertension and dyslipidemia on the decline in renal function. Hypertension.

[11] Bushinsky DA, Monk RD (1998). Electrolyte quintet: Calcium. Lancet.

[12] Nair CG, Babu M, Jacob P, Menon R, Unnikrishnan MJ (2016). Renal dysfunction in primary hyperparathyroidism; effect of parathyroidectomy: A retrospective cohort study. Int J Surg.

[13] Mitchell DM (2012). Long-term follow-up of patients with hypoparathyroidism. J Clin Endocrinol Metab.

[14] Daimon M (2016). Association Between Pituitary-Adrenal Axis Dominance Over the Renin-Angiotensin-Aldosterone System and Hypertension. J Clin Endocrinol Metab.

[15] Kamba A (2016). Association between Higher Serum Cortisol Levels and Decreased Insulin Secretion in a General Population. PLoS One.

[16] Kidney disease: improving global outcomes (KDIGO) (2013). Chapter 1: Definition and classification of CKD. Kidney International Supplements (2011).

[17] Fleming JS, Zivanovic MA, Blake GM, Burniston M, Cosgriff PS (2004). British Nuclear Medicine Society. Guidelines for the measurement of glomerular filtration rate using plasma sampling. Nucl Med Commun.

[18] Pottel H (2017). Age-dependent reference intervals for estimated and measured glomerular filtration rate. Clin Kidney J.

[19] Payne RB, Little AJ, Williams RB, Milner JR (1973). Interpretation of serum calcium in patients with abnormal serum proteins. Br Med J.

[20] Matsuo S (2009). Collaborators developing the Japanese equation for estimated GFR. Revised equations for estimated GFR from serum creatinine in Japan. Am J Kidney Dis.

[21] Committee of the Japan Diabetes Society on the Diagnostic Criteria of Diabetes Mellitus, Seino Y (2010). Report of the committee on the classification and diagnostic criteria of diabetes mellitus. J Diabetes Investig.

[22] Peacock M (2010). Calcium metabolism in health and disease. Clin J Am Soc Nephrol.

[23] Clark WF (2011). Urine volume and change in estimated GFR in a community-based cohort study. Clin J Am Soc Nephrol.

[24] Hebert LA, Greene T, Levey A, Falkenhain ME, Klahr S (2003). High urine volume and low urine osmolality are risk factors for faster progression of renal disease. Am J Kidney Dis.

[25] Moe SM, Drüeke T, Lameire N, Eknoyan G (2007). Chronic kidney disease-mineral-bone disorder: a new paradigm. Adv Chronic Kidney Dis.

[26] Li YC (2010). Renoprotective effects of vitamin D analogs. Kidney Int.

[27] Li YC (2002). 1,25-Dihydroxyvitamin D(3) is a negative endocrine regulator of the renin-angiotensin system. J Clin Invest.

[28] Durvasula RV, Shankland SJ (2006). The renin-angiotensin system in glomerular podocytes: mediator of glomerulosclerosis and link to hypertensive nephropathy. Curr Hypertens Rep.

[29] Panichi V (1998). Calcitriol modulates *in vivo* and *in vitro* cytokine production: a role for intracellular calcium. Kidney Int.

[30] Kuhlmann A (2004). 1,25-Dihydroxyvitamin D3 decreases podocyte loss and podocyte hypertrophy in the subtotally nephrectomized rat. Am J Physiol Renal Physiol.

[31] Schwarz U (1998). Effect of 1,25 (OH)2 vitamin D3 on glomerulosclerosis in subtotally nephrectomized rats. Kidney Int.

[32] de Boer IH (2011). Serum 25-hydroxyvitamin D and change in estimated glomerular filtration rate. Clin J Am Soc Nephrol.

